# The effects of postoperative targeted immunotherapy on peripheral blood cytokines and immune cell profile in lung cancer patients

**DOI:** 10.3389/fonc.2024.1342624

**Published:** 2024-06-05

**Authors:** Chuang Zhang, Hongmei Mo, Min Li, Shuaiyan Wang, Xiaowen Dou, Xiuming Zhang

**Affiliations:** ^1^ School of Medicine, Anhui University of Science and Technology, Huainan, China; ^2^ Medical Laboratory of the Third Affiliated Hospital of Shenzhen University, Shenzhen, China; ^3^ Department of Laboratory Medicine, Clinical Medical College of Yangzhou University, Subei People’s Hospital of Jiangsu Province, Yangzhou, China

**Keywords:** lung cancer, cytokines, cell subsets, immunotherapy, tumor progression

## Abstract

**Objective:**

Cytokines and cell subsets are important components of the tumor microenvironment. Previous research has revealed that there are differences in cytokines and cell subsets in the peripheral blood of lung cancer (LCA) patients before and after eradication. The purpose of this study is to explore the monitoring value of cytokines and cellular subpopulations as biomarkers in post-immunotherapy monitoring of patients with LCA after surgery

**Methods:**

We conducted a case-control study using double-antibody sandwich magnetic microsphere flow cytometry with immunofluorescence technology and fluorescent monoclonal antibody multiparameter flow cytometry to detect differences in peripheral blood cytokines and cell subsets between LCA patients after immunotherapy and healthy controls.

**Results:**

Our research results show that there are differences in the levels of IL-4, IL-6, IL-10, IL-17, IFN-γ, TNF-α in the peripheral blood of LCA patients (n=70) after immunotherapy compared to the healthy controls (n=55) (*P<0.05*), and there are differences in 10 cell subgroups including DP T Cells, AT cells, and NLR in the peripheral blood compared to the healthy controls (n=35) (*P<0.05*). Further analysis revealed significant differences in the detection data of IL-6, IL-10, IFN-γ, CD56^dim^ NK cells, Total B cells, Total NE cells, CD15^+^M cells, and NLR between LCA deceased patients (n=25) and LCA surviving patients (n=27) during the same period (*P<0.05*). The continuous monitoring of cytokines and cell subsets is far more valuable than a single-time test, as abnormal fluctuations in the data of cytokines and cell subsets are often associated with poor prognosis. In addition, IL-6 and NLR showed the strongest discriminative ability between postoperative immunotherapy-treated LCA patients and healthy controls, with AUC values of 0.840 and 0.822, respectively. There was a significant association between IFN-γ and distant metastasis in LCA (*P<0.05*), as well as between CD56^dim^ NK cells and lymph node infiltration (*P<0.05*).

**Conclusion:**

This research results support peripheral blood cytokines and cell subsets as biomarkers for monitoring the postoperative immune status and predicting the prognosis of LCA patients after immunotherapy. The continuous monitoring of cytokines and cell subsets is far more valuable than a single-time detection.

## 1 Introduction

Lung cancer (LCA) is a major contributor to cancer-related morbidity and mortality (1). Approximately 2.1 million new cases of LCA are diagnosed worldwide each year, with over 1.8 million deaths occurring annually (2). The current treatment strategy for sufficiently healthy LCA patients primarily revolves around surgical resection, supplemented by radiation therapy, chemotherapy, and the latest targeted immunotherapy (3). As the types of immunotherapy for LCA, such as therapeutic vaccines, immune modulators, autologous cell therapy, and monoclonal antibodies targeting checkpoint inhibitors signaling associated with activated T cells and/or cancer cells, continue to increase, the reliability and effectiveness of targeted immunotherapy have been validated (4). However, due to the individual variability among LCA patients and the uniqueness of each immune therapy’s target, the responsiveness of LCA patients to immunotherapy varies greatly (5). Therefore, a biomarker that can be repeatedly tested is needed to assess the immune status of LCA patients, evaluate treatment outcomes, and provide information for treatment decisions. Traditional LCA tumor markers, such as Carbohydrate Antigen 125 (CA125) and Neuron-Specific Enolase (NSE), are believed to be useful for monitoring the efficacy of immunotherapy in LCA patients. However, multiple studies have found that these tumor markers may not accurately reflect the treatment response of lung cancer patients (6, 7). In recent years, liquid biopsy markers such as Circulating Tumor Cells (CTC), Cell-Free DNA (cfDNA), and Exosomes have been considered as potential biomarkers for predicting immune efficacy after LCA immunotherapy. However, tumor heterogeneity and the high cost of detection may limit the clinical application of this technology (8). Peripheral blood cytokines and immune cells are considered intriguing targets for immunotherapy and clinical biomarker research, as they can reflect changes in the tumor microenvironment during treatment, patient treatment responsiveness, and provide crucial information about the progression of LCA disease (8–11). Compared to the tumor tissue obtained through surgery, which can only undergo immunoscore testing once (12), peripheral blood samples can be collected throughout the entire treatment of LCA patients, enabling dynamic monitoring of peripheral blood cytokines and immune cells (13, 14).

The peripheral blood contains various cytokines and immune cells, which play a particularly crucial role in the occurrence, prognosis, and treatment of LCA (15–17). Inflammatory cytokines are associated with the advanced stages of LCA, resistance to immunotherapy, and poor prognosis, such as tumor necrosis factor-α (TNF-α), interleukin (IL)-8 increase with the progression of late-stage small cell LCA (18). IL-6 is an important cytokine in LCA that promotes epithelial-mesenchymal transition and tumor metastasis, facilitating the migration and invasion of LCA cells (19). The expression of the immunosuppressive cytokine IL-10 is correlated with the survival outcomes and treatment response rates of LCA patients. Multiple research findings indicate that patients whose serum IL-10 remained high levels during treatment have a worse prognosis (20). Interferon γ (IFN-γ) plays multiple roles in tumor immune responses. IFN-γ produced after the activation of T lymphocytes by tumor antigens can stimulate the proliferation and differentiation of tumor-infiltrating lymphocytes. It can also generate immunosuppressive factors, leading to direct negative feedback regulation of effector T cell function (21). Peripheral blood immune cells can dynamically reflect changes in the tumor microenvironment, the outcomes of anti-tumor immune responses, and predict clinical responses to immunotherapy (22), such as T-cell exhaustion associated with surgical complications and higher mortality rates, which can reflect the clinical treatment status (23). Natural killer (NK) cells, as a type of innate immune cell, can attack tumor cells without the need for activation (24). NK cells can be divided into CD56^dim^ cell subsets, which express cytotoxicity, and CD56^bright^ cell subsets, which primarily produce cytokines, based on the expression of CD56 (25). The special T-cell subset that simultaneously expresses the TCR receptor and CD56 receptor is called NKT cell subset, mainly involved in regulating the body’s responses to infections, tumor immunity, immune surveillance, and the perforin and granule enzymes expressed in their lytic granules can lyse tumor cells (26). The neutrophil-to-lymphocyte ratio (NLR) is associated with improved progression-free survival and overall survival in LCA patients (27).

Our hospital employs peripheral blood immune cells and a combination of seven cytokines for monitoring the immune status of LCA patients undergoing immunotherapy. However, due to the use of a clinical test kit commonly applicable to all cancers, it does not specifically indicate effectiveness for LCA, and there is also a lack of relevant research in this area. The study utilized flow cytometry to analyze changes in cytokines and immune cell phenotypes in the peripheral blood circulation of patients with LCA after targeted immunotherapy, and its correlation with the clinical characteristics of LCA patients. The aim was to comprehensively evaluate the value of using cytokines and cell subsets in postoperative immune monitoring of LCA patients and identify meaningful biomarkers for testing.

## 2 Materials and methods

### 2.1 Study population

The study population included patients who underwent lung tumor resection surgery at the Luohu Hospital Group (Shenzhen, China) between January 2019 and December 2022 and were diagnosed with LCA. The inclusion criteria were patients with LCA who underwent R0 resection surgery and had histopathological confirmation. The exclusion criteria were patients with concurrent other cancers or immune-related diseases, those who received preoperative radiotherapy or chemotherapy, or those who had used immunomodulatory drugs in the past three years. Peripheral blood cytokine and immune cell testing data included all data from the first postoperative test for each patient until the end of the data collection period, with each patient receiving at least one peripheral blood cytokine and immune cell test after immunotherapy. Due to the limited number of patients undergoing simultaneous testing of peripheral blood cytokines and immune cell populations during their physical examinations, we selected 55 physical examination patients who were tested for cytokines at the same time and 35 physical examination patients who were tested for cell subsets as healthy controls. The specimens from LCA patients after immunotherapy were collected from those who received immunotherapy immediately after surgery for more than five months (average 9.4 months, range 5.0-18.5 months). The time of specimen collection for LCA patients before death is the time of the last specimen collection before the death report is issued (average 1.7 months, range 0.1-8.1 months). In addition, clinical data of patients with LCA were also collected.

### 2.2 Analysis of peripheral blood cytokines

The plasma separation method for LCA patients involves using peripheral blood samples with EDTA anticoagulant, centrifuging at 1500g for 15 minutes, which can be used for immediate experiments or stored at -20°C. Add 50 μL of capture microsphere suspension and 50 μL of plasma sample to the centrifuge tube, mix thoroughly, and incubate at room temperature in the dark for 1 hour. Perform magnetic separation to remove the supernatant, then add 100 μL of fluorescently labeled antibody working solution, mix thoroughly, and incubate at room temperature in the dark for 1 hour. Perform magnetic separation again to remove the supernatant, and wash the microspheres with 200 μL of magnetic bead dilution solution; repeat this step twice. Finally, resuspend the microspheres in 200 μL of magnetic bead dilution solution and detect using the EasySample flow cytometer. The reagents and equipment were purchased from Wellgrow Company (Shenzhen, China).

### 2.3 Analysis of peripheral blood immune cell profile

Add 5μL of labeled antibody to 100μL of EDTA anticoagulated blood, mix well, and incubate at room temperature in the dark for 15 minutes. Add 200-300μL of red blood cell lysis solution, mix well, and incubate in the dark for 10-15 minutes. Add 1.5ml of flow cytometry sheath fluid, mix, centrifuge at 400g for 5 minutes, discard the supernatant, and repeat this step twice. Finally, add 300μL of sheath fluid for Beckman Navios flow cytometry analysis. The cell concentration should not exceed 5*10^9^. Based on the characteristics of cellular immune phenotypes, four multi-color flow cytometry panels were designed to identify immune cell subsets (**Supplementary Table 1**). The reagents and instruments were purchased from Beckman Coulter, Inc. (Brea, California, USA).

### 2.4 Flow cytometry data analysis

Use the operating software of the EasySample flow cytometer to analyze the cytokine dataset; use Kaluza Analysis software to analyze the dataset of cell subsets. The EasySample flow cytometer prepares a quantitative calibration standard (**Supplementary Table 2**) and constructs a standard curve before each use to ensure the comparability of instrument performance at different times. The Beckman Navios flow cytometry requires the addition of a control tube (isotype control antibody) each time a sample is tested, and daily instrument quality control is performed using the Cell Sorter & Tracker (CS&T) application to ensure that the detection results do not change over time. The above quality control materials are reagent accessories provided by a reagent company.

### 2.5 Statistical analyses

Statistical analysis was performed using SPSS software (IBM SPSS Statistics 26, Chicago, USA). In appropriate circumstances, use independent sample T-test and Mann-Whitney U-test to compare the differences in peripheral blood cytokines and cell subsets between LCA patients and healthy donors. In addition, Pearson χ2 test was used to analyze the correlation between certain cytokines, immune cells, and clinical features of tumors. Figures were created by GraphPad Prism 9.0. *P*-values ≤ 0.05 were considered statistically significant.

## 3 Result

### 3.1 Patient characteristics

This study included a total of 70 LCA patients with data from peripheral blood cytokine and immune cell subset tests, as well as data from 55 healthy control individuals for cytokine testing and 35 healthy control individuals for immune cell subset testing. By the end of data retrieval, 25 out of the 70 LCA patients (35.71%) had passed away. The LCA patients were predominantly in the advanced stage, with 54 out of 70 (77.1%) having tumor grading primarily at stage IV, 39 out of 70 (55.7%) having poorly differentiated tumors, and distant lymph node metastasis and distant metastasis occurring in 57 out of 70 (81.4%) and 12 out of 70 (17.1%) cases, respectively. The age of LCA patients showed a higher trend compared to that of the healthy controls. **Table 1** summarizes the clinical and pathological characteristics of LCA patients and healthy controls.

**Table 1:** Patient demographics and tumor characteristics.

	LCA patients	Cytokines healthy donors	Cell subset healthy donors
	(N=70)	(N=35)	(N=55)
Age*			
Median (years)	65	55	56
Range (years)	32-79	32-73	33-78
Sex			
Male	47 (67.1%)	25 (71.4%)	36 (65.5%)
Female	23 (32.9%)	10 (28.6%)	19 (34.5%)
Tumor stage			
Stage I	1 (1.4%)		
Stage II	4 (5.7%)		
Stage III	7 (10.0%)		
Stage IV	54 (77.1%)		
Unknown	4 (5.7%)		
Tumor differentiation grade			
Well/moderate	22 (31.4%)		
Poor	39 (55.7%)		
Unknown	9 (12.9%)		
Lymph node invasion			
Yes	57 (81.4%)		
No	6 (8.6%)		
Unknow	7 (10%)		
Neoadjuvant radiotherapy			
Yes	12 (17.1%)		
No	58 (82.9%)		
Adjuvant chemotherapy			
Yes	65 (91.9%)		
No	5 (7.1%)		
Survival state			
Living	45 (64.29%)		
Dying	25 (35.71%)		

*Age at time of surgery.

### 3.2 Increase in six cytokine levels in the plasma of LCA patients compared to healthy individuals

In this section of the study, we compared the differences in levels of 7 cytokines (IL-2, IL-4, IL-6, IL-10, IL-17, IFN-γ, TNF-α) in peripheral blood plasma between LCA patients after targeted immunotherapy and healthy controls (**Table 2**). The levels of IL-2(P* = 0.367) were similar in LCA patients and healthy individuals, while the expression levels of IL-4 (P*=0.037), IL-6 (P* < 0.001), IL-10 (P* = 0.020), IL-17 (P* = 0.010), IFN-γ (P* = 0.018), and TNF-α (P* = 0.030) were increased.

**Table 2:** Comparison between peripheral Cytokines in lung cancer patients and healthy controls.

	Healthy donors (n=55)		LCA patients (n=70)		P*-value
	Mean	SD	Mean	SD	
IL-2	0.81	1.06	3.67	16.29	0.367
IL-4	0.67	0.83	2.15	7.12	**0.037**
IL-6	2.93	3.22	32.98	93.75	**< 0.001**
IL-10	1.57	1.36	3.96	10.66	**0.020**
IL-17	1.39	2.30	3.15	10.95	**0.010**
IFN-γ	2.28	3.85	7.69	12.91	**0.018**
TNF-α	0.59	0.66	3.16	5.74	**0.030**

*Mann-Whitney U test. P* value ≤ 0.05 were considered statistically significant and are indicated in bold.

### 3.3 Differences in the percentages of 11 immune cell types and NLR between LCA patients and healthy controls

**Figure 1:**
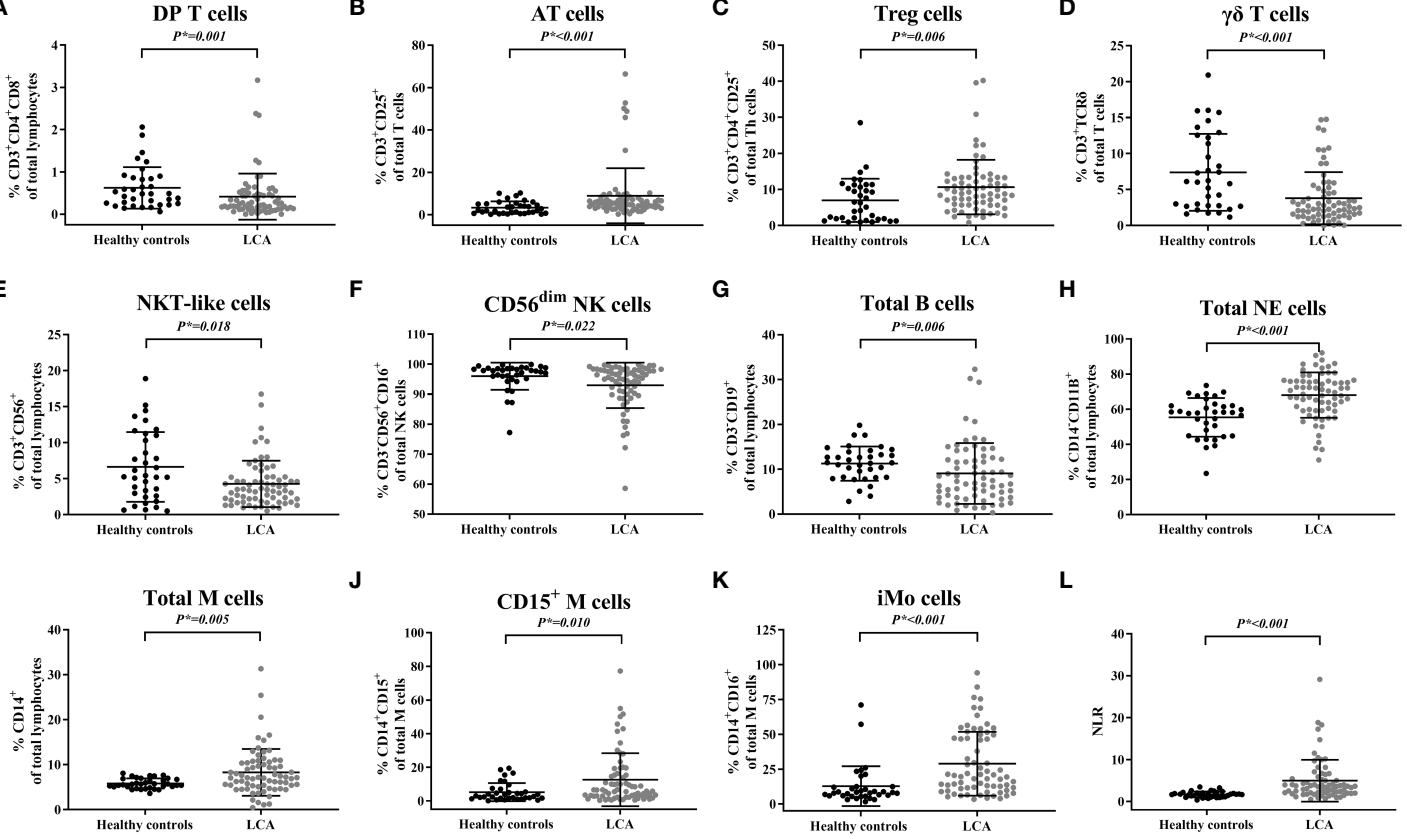
The peripheral blood immune cell subset distribution in lung cancer patients compared to healthy controls. **(A)** The percentage of DP T cells. **(B)** Percentage of AT cells. **(C)** Percentage of treg cells. **(D)** Percentage of γδ T cells. **(E)** Percentage of NKT-like cells. **(F)** Percentage of CD56dim NK cells. **(G)** Percentage of total B cells. **(H)** Percentage of total NE cells. **(I)** Percentage of total M cells. **(J)** Percentage of CD15+ M cells. **(K)** Percentage of iMo cells. **(L)** Values of NLR.

Afterwards, we assessed the differences in peripheral blood immune cell profiles between LCA patients following immunotherapy and healthy controls (**Table 3**). In the T cell subsets, the percentage of double-positive T cells (DP T Cells/CD3^+^CD4^+^CD8^+^) (P* = 0.001) and γδ T cells (CD3^+^TCRγδ^+^%) (P* < 0.001) were decreased (**Figures 1A, D**), while the percentage of activated T cells (AT Cells/CD3^+^CD25^+^) (P* < 0.001) and regulatory T cells (Treg Cells/CD3^+^CD4^+^CD25^+^) (P* = 0.006) were increased (**Figures 1B, C**). The percentage of a specific T cell subset, NKT-like cells (CD3^+^CD56^+^), was also decreased (**Figure 1E**). The percentage of CD56^dim^ NK cells (CD3^-^CD56^+^CD16^+^) in the NK cell subset was comparable between LCA patients and healthy individuals (P* = 0.022) (**Figure 1F**). The percentage of total B cells (CD3^-^CD19^+^) in lymphocytes is decreased (P* = 0.006), while the percentage of total neutrophils (NE/CD14^-^CD11B^+^) and total monocytes (M/CD14^+^) is increased (P* < 0.001 and P* = 0.005, respectively) (**Figures 1G–I**). Meanwhile, the CD15^+^ monocytes (CD14^+^CD15^+^) and intermediate monocytes (iMo/CD14^+^CD16^+^) in mononuclear cells both increased (P* = 0.010 and P* < 0.001, respectively) (**Figures 1J, K**). The NLR of LCA patients is elevated compared to healthy controls (P* < 0.001) (**Figure 1L**).

**Table 3:** Comparison between peripheral blood immune cell profiles in lung cancer patients and healthy controls.

	Healthy donors (n=35)		LCA patients (n=70)		P*-value
	Mean	SD	Mean	SD	
CD3^+^ (%)	67.06	9.04	63.14	12.53	0.126^U^
CD3^+^CD4^+^ (%)	39.15	8.10	36.57	9.73	0.183^T^
CD3^+^CD8^+^ (%)	24.63	7.51	24.56	9.01	0.967^T^
CD3^+^CD4^+^/CD3^+^CD8^+^	1.76	0.70	1.71	0.85	0.529^U^
CD3^+^CD4^+^CD8^+^ (%)	0.62	0.48	0.41	0.54	**0.001^U^**
CD3^+^CD25^+^ (%)	3.32	2.90	8.95	12.92	**< 0.001^U^**
CD3^+^CD4^+^CD25^+^ (%)	6.99	5.92	10.65	7.50	**0.006^U^**
CD3^+^CD8^+^CD25^+^ (%)	1.40	2.03	7.47	22.19	0.097^U^
CD3^+^CD56^+^ (%)	6.63	4.77	4.27	3.20	**0.018^U^**
CD3^-^CD56^+^ (%)	14.52	8.61	12.91	7.28	0.421^U^
CD3^-^CD56^+^CD16^+^ (%)	96.01	4.46	91.90	11.52	**0.022^U^**
CD3^+^CD56^+^CD16^+^CD57^+^ (%)	52.49	19.93	49.04	15.13	0.330^T^
CD3^+^TCRγδ^+^ (%)	9.22	11.85	3.80	3.60	**< 0.001^U^**
CD3^+^TCRγδ^+^CD25^+^ (%)	2.77	4.41	5.74	10.65	0.296^U^
CD3^-^CD19^+^ (%)	11.27	3.76	9.07	6.74	**0.006^U^**
CD3^-^CD19^+^CD25^+^ (%)	11.72	15.48	11.42	13.58	0.126^U^
CD14^-^CD11B^+^ (%)	55.36	10.84	68.02	12.81	**< 0.001^T^**
CD14^-^CD11B^+^CD15^+^ (%)	88.87	13.35	90.56	13.20	0.473^U^
CD14^-^CD11B^+^CD16^+^ (%)	91.21	6.39	90.69	11.78	0.480^U^
CD14^+^ (%)	5.82	1.11	8.29	5.16	**0.005^U^**
CD14^+^CD15^+^ (%)	5.21	5.30	12.62	15.62	**0.010^U^**
CD14^+^CD16^+^ (%)	12.84	14.11	28.93	22.73	**< 0.001^U^**
NLR	1.69	0.69	4.97	4.94	**< 0.001^U^**

* ^T^ Independent samples T test;^U^ Mann-Whitney U test. P* value ≤ 0.05 were considered statistically significant and are indicated in bold.

### 3.4 Elevated levels of IL-6, IL-10, and INF-γ before death in targeted immunotherapy for LCA patients

**Figure 2:**
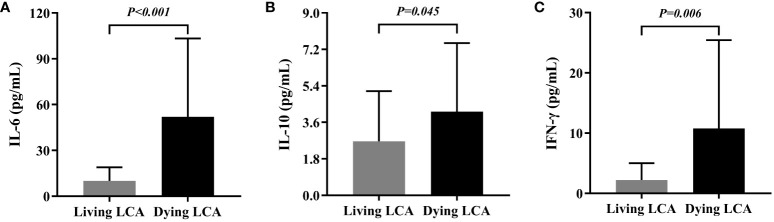
Comparison between peripheral Cytokines in living lung cancer patients and dying lung cancer. **(A)** Differential expression of IL-6 in living lung cancer patients and dying lung cancer. **(B)** Differential expression of IL-10 in living lung cancer patients and dying lung cancer. **(C)** Differential expression of IFN-γ in living lung cancer patients and dying lung cancer.

In this section, we examined the differences in IL-4, IL-6, IL-10, IL-17, IFN-γ, and TNF-α values between 25 dying LCA patients’ last test and 27 LCA patients who were alive during the same period (**Supplementary Table 3**). The average survival time of dying LCA patients was 11.9 months, with a median of 10.2 months; therefore, the control group (survivors) specimen collection time was chosen as 10-12 months post-surgery. The levels of IL-4, IL-17, and TNF-α were similar in the plasma of LCA dying and living patients, while the levels of IL-6, IL-10, and IFN-γ were elevated in the plasma of LCA dying patients (**Figures 2A–C**). Further analyze the dynamic changes of plasma cytokines in LCA patients after targeted immunotherapy. Continuous monitoring curves were performed on 6 dying LCA patients who had been tested for cytokines more than 5 times and 10 living LCA patients, and the results are shown in **Supplementary Figure 1**. Additionally, due to independent IL-6 monitoring, there were two extra patients in both the dying and living LCA groups, totaling eight and twelve patients, respectively. In LCA patients, we observed an increased trend in the levels of certain plasma cytokines prior to death. For example, we observed elevated levels of IL-6 and IL-10 before death in five out of six LCA patients, and elevated levels of IFN-γ before death in four out of six LCA patients. However, the expression levels of plasma cytokines in living LCA patients remained relatively stable, even gradually decreasing. Only a few patients showed elevated levels, namely IL-6 (3/12), IL-10 (2/10), and IFN-γ (2/10).

### 3.5 Differences in peripheral blood cell subsets between dying and living LCA patients

**Figure 3:**
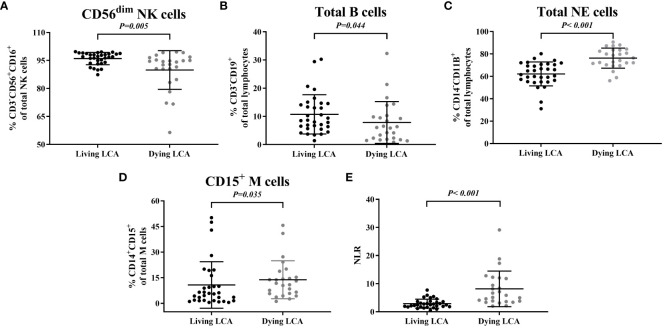
Comparison between peripheral blood immune cell profiles in living lung cancer. **(A)** Percentage of CD56dim NK cells. **(B)** Percentage of total B cells. **(C)** Percentage of total NE cells. **(D)** Percentage of CD15+ M cells. **(E)** Values of NLR.

Subsequently, we selected the immune cells that had previously been confirmed to exhibit differences between LCA patients and healthy individuals in the earlier part of the study and investigated their differences between dying and living LCA patients (**Supplementary Table 4**). The percentage of CD56^dim^ NK cells in NK cells decreased, as did the percentage of Total B cells in lymphocytes (*P* = 0.005 and *P* = 0.044, respectively) (**Figures 3A, B**). Additionally, the percentage of Total NE cells in lymphocytes increased, as did the percentage of CD15^+^M cells in monocytes (*P* < 0.001 and P = 0.035, respectively) (**Figures 3C, D**). Furthermore, compared to living LCA patients, NLE significantly increased in LCA patients before death (*P* < 0.001) (**Figure 3E**). Similarly, select LCA patients who have undergone testing more than 5 times to plot continuous monitoring curves, investigating the dynamic trends in the percentage of cell subsets. A total of 7 LCA dead patients and 11 LCA alive patients met the requirements. Among the three critically dying LCA patients, there was a declining trend in the percentage of CD56dim NK cells, with one individual experiencing a sharp decline followed by a brief recovery. In contrast, among the eleven living LCA patients, the percentage of CD56^dim^ NK cells fluctuated within a narrower range overall, with smaller overall variations compared to the critically dying LCA patients (**Supplementary Figure 2A, B**). The percentage of Total B cells gradually decreased in 5 cases of LCA dying patients, while the percentage of Total B cells remained relatively stable in 11 cases of LCA living patients, with no significant change in trend (**Supplementary Figure 2C, D**). Among the 7 dying LCA patients, there was a trend of increased total NE cell percentage before death, while the 11 surviving LCA patients showed interval fluctuations without a significant upward trend change (**Supplementary Figures 2E, F**). Among the 7 critically dying LCA patients, only two showed a relatively stable percentage of CD15^+^M cells before death; whereas among the 11 living patients, 7 exhibited relative stability (**Supplementary Figures 2G, H**). In both critically dying and living LCA patients, no clear trend change was observed in NLR, but the NLR extremes in critically dying LCA patients were higher than in living LCA patients (**Supplementary Figure 2I, J**).

### 3.6 Association between peripheral blood cytokines, cell subsets and LCA patient clinical features

**Figure 4:**
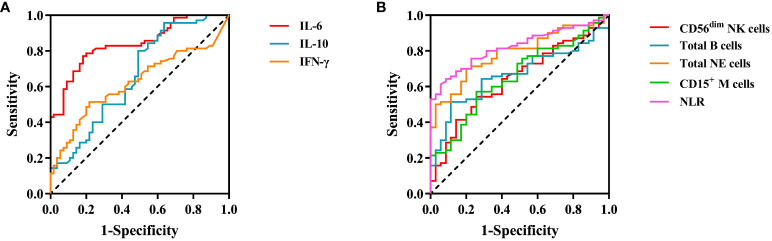
ROC curve for peripheral blood cytokines and immune cell in lung cancer. **(A)** Cytokines. **(B)** Immune cell.

After the research revealed differences in three cytokines and five cell subgroups between LCA patients and healthy controls, as well as between LCA dying patients and living patients, we studied their correlation with tumor characteristics. First, we analyzed the diagnostic capabilities of eight indicators, including three cytokines and five cell subsets, between LCA patients and healthy controls (**Figure 4**). Then, based on the parameters of the ROC curve, we calculated various indicators such as the Youden index, area under curve (AUC) area, sensitivity, and specificity for each indicator to identify the most suitable cutoff values for each indicator (**Table 4**). The AUC area for IL-6 and NLR are relatively high, with *P* < 0.001. IL-6 exhibits high sensitivity and specificity at the cutoff, while NLR has slightly lower sensitivity but the highest specificity. Both of them demonstrate strong discriminative ability between LCA patients and healthy individuals. Therefore, IL-6 and NLR may be effective biomarkers for postoperative immune therapy outcomes and patient prognosis in LCA patients.

**Table 4:** ROC and cutoff values analysis of cytokines and immune cell in lung cancer patients after immunotherapy.

	Area under Curve	Youden index	Sensitivity	Specificity	Cutoff (pg/mL)	*P-value*
IL-6	0.840 (0.772-0.908)	0.59	0.78	0.82	4.02	< 0.001
IL-10	0.654 (0.555-0.753)	0.32	0.96	0.36	0.57	0.002
IFN-γ	0.623 (0.525-0.721)	0.30	0.51	0.78	2.30	0.019
CD3^-^CD56^+^CD16^+^ (%)	0.643 (0.534-0.751)	0.29	0.54	0.74	95.87	0.018
CD3^-^CD19^+^ (%)	0.665 (0.562-0.768)	0.40	0.51	0.89	7.60	0.006
CD14^-^CD11B^+^ (%)	0.787 (0.702-0.872)	0.50	0.70	0.80	63.43	< 0.001
CD14^+^CD15^+^ (%)	0.655 (0.548-0.702)	0.31	0.57	0.74	5.45	0.010
NLR	0.822 (0.745-0.900)	0.56	0.64	0.91	2.62	< 0.001

The differences in three cytokines and five cell subset markers were separately investigated in tumor grading, differentiation, lymph node infiltration, and distant metastasis (**Supplementary Tables 5, 6**). Only IFN-γ showed a difference in distant metastasis (*P* = 0.002), and CD3^-^CD56^+^CD16^+^ (%) showed a difference in lymph node infiltration (*P* = 0.028). In addition to these findings, we did not observe any correlation between other cytokines, immune cell subsets, and tumor characteristics.

## 4 Discussion

Recently, some studies using the Immunoscore system to monitor the abundance of immune cells in cancer patient tumor tissues, predict survival outcomes, and assess immune therapy responses have confirmed the ability of the immune status of cancer patients as a predictive biomarker for tumor immunotherapy (28–30). The high infiltration of tumor immune cells and a higher score for the immune microenvironment are associated with favorable immune therapy efficacy and clinical outcomes in LCA (31). However, due to the significant trauma associated with tumor tissue biopsies and the insufficient repeatability of specimen collection, dynamic monitoring of the immune status of tumor patients cannot be achieved solely based on a single surgical specimen (32). The peripheral blood sample is easy to obtain and causes minimal trauma, making it very suitable for monitoring the immune function status of cancer patients dynamically (33). Over the years, despite the standard treatment strategies for LCA remaining surgery, chemotherapy, and radiation therapy, in recent years, tumor immunotherapy aimed at stimulating the host’s own immune system to eradicate cancer has been gradually gaining attention (34). In order to elucidate the correlation between the immune status of LCA patients after immunotherapy and disease progression, this study characterized the levels of peripheral blood cytokines and the distribution of cell subsets. We explored the differences in these cytokines and immune cell subsets between LCA patients and healthy individuals, as well as their varying changes in tumor patients with different responses to immunotherapy.

We observed changes in the peripheral blood cell cytokines in LCA patients after postoperative immunotherapy. Compared to healthy individuals, we found that the levels of IL-4, IL-6, IL-10, IL-17, IFN-γ, and TNF-α in the plasma increased in LCA patients after immunotherapy following surgery, consistent with previous studies (21, 35–38). The high levels of IL-4 can induce an increase in the activity of tissue proteases in macrophages, thereby promoting tumor growth, invasion, and tumor angiogenesis (39). In addition, the excessive production of vascular endothelial growth factor (VEGF) is positively correlated with elevated levels of IL-4, indicating that IL-4 may support tumor progression through different mechanisms (40). Patients with lower baseline IL-6 levels after immunotherapy are more likely to benefit from immunotherapy. This is consistent with a previous conclusion regarding PD-L1 treatment in small cell LCA (41). Research has shown that IL-10 inhibits the PD-1/PD-L1 pathway, leading to resistance of tumors to PD-1/PD-L1 immunotherapy. This suggests that IL-10 may have adverse effects in immunotherapy and also explains why individuals with higher levels of IL-10 in this study had a worse prognosis (42). A clinical retrospective study suggests that during treatment, the levels of IL-17 are associated with clinical benefits of immunotherapy, but are unrelated to baseline levels in peripheral blood before treatment (43). IFN-γ, on one hand, promotes the production of immunosuppressive molecules, thereby exerting direct negative feedback on the function of effector T cells. On the other hand, during the elimination phase of the tumor immune response, elevated levels of IFN-γ increase the cytotoxic activity against LCA tumor cells, and this mechanism of action leads to autoimmune-like side effects (43, 44). TNF-α plays a complex role in tumor immunity, often used as a tool to regulate immune cells and kill tumor cells. However, prolonged exposure to high concentrations of TNF-α levels can instead promote tumor progression and may lead to hemorrhagic necrosis (45). To further elucidate the value of cytokines in the dynamic monitoring of the immune status in LCA patients, we conducted additional analyses of changes in cytokines after postoperative immunotherapy. We compared the peripheral blood cytokines of LCA patients who died after immunotherapy with those of LCA patients who were alive during the same period. The average levels of IL-4, IL-6, IL-10, IL-17, IFN-γ, and TNF-α before the death of LCA patients were elevated, with statistically significant differences observed in IL-6, IL-10, and IFN-γ. Long-term surviving LCA patients often exhibit stable changes in plasma cytokine levels, while cytokine levels in deceased patients show a gradual or severe increase before death. Recent studies have also reached consistent conclusions, such as elevated IL-6 levels, suggesting a poor response to pembrolizumab therapy in advanced renal cell carcinoma treatment, as well as worse survival outcomes (46). Interrupting IL-10 signal transduction can induce the death of tumor cells in PD-1 treatment-resistant patients with colorectal cancer liver metastasis and activate the immune system’s anti-tumor response (47). The appropriate level of cytokines has a positive effect on the anti-tumor treatment of cancer patients, but high levels of cytokines often promote tumor development instead (37). Our research results indicate that a sharp increase in cytokines may be associated with a poor prognosis in LCA patients, while relatively stable cytokine levels are often associated with a longer survival period in LCA patients.

Our research has found differences in the percentages of cell subsets between LCA patients and healthy controls, and these findings are consistent with previous studies in multiple tumor research. DP T cells are a multifunctional T cell subset derived from single-positive T cells (SP T cells) following antigen stimulation, and their function may be related to the ability to enhance T cell memory levels (48). In this study, the percentage of DP T cells in LCA patients is decreased, which is inconsistent with the results of studies on other cancers, and further cohort validation of this result is needed in the future (49). Compared to healthy individuals, LCA patients have a lower percentage of peripheral blood γδ T cells and NKT-like cells (50, 51), and a higher percentage of AT cells, Treg cells, and Total M cells (52–54). These findings have been reported in multiple cancer studies. In addition, we found that the percentages of CD56^dim^ NK cells, Total B cells, Total NE cells, CD15^+^ M cells, and the NLR not only showed differences between LCA patients and healthy individuals but also exhibited differential characteristics within the cell subsets tested concurrently in LCA deceased patients and surviving patients. In this study, the percentage of CD56^dim^ NK cells is inversely correlated with the course of cancer in LCA patients, indicating a negative association between the levels of CD56^dim^ NK cells after immunotherapy and the prognosis of LCA patients. CD56^dim^ NK cells are primarily involved in cell lysis and target cell killing, while also being a significant source of pro-inflammatory and chemotactic factors. Therefore, a decrease in the levels of CD56^dim^ NK cells leads to a reduced ability of LCA patients to eliminate tumor cells (55). More and more evidence suggest that B cells play a crucial synergistic role in tumor control. A decrease in the overall levels of B cells leads to a reduced B cell-mediated antigen presentation, diminished capacity of the body to produce cytokines, decreased antibody-dependent cellular cytotoxicity, and reduced phagocytic activity (56). As the most abundant cell type in the human body, neutrophils serve as important regulators of cancer. The elevation of total NE cells levels is closely associated with cancer development, tumor angiogenesis, and tumor immune suppression. Previous studies have confirmed the increase in neutrophil levels in various types of tumors (57). The clinical research on CD15^+^ monocytes in LCA is limited, considering that CD15^+^ is a characteristic marker of monocyte differentiation, it may be related to the anti-tumor chemotactic activity of monocytes (58). A study on the correlation between NLR and LCA prognosis found that the NLR values of patients who died during the follow-up period significantly increased, while the NLR values of patients who were still alive remained stable. There was a significant correlation between NLR values and the follow-up time for both LCA patients who died and those who survived, and their conclusions align with ours (59). In summary, the changes in the percentage of LCA cell subsets are correlated with the post-immunotherapy immune status of LCA patients. Continuous monitoring of changes in cell subsets can provide a better understanding of the immune function and therapeutic efficacy of LCA patients and assist in predicting the prognosis of LCA patients.

We also compared the discriminative abilities of cytokines and cell subsets after immunotherapy between LCA patients and healthy controls. IL-6 and NLR showed the best performance, with AUC values of 0.840 and 0.822, respectively. This suggests that IL-6 and NLR have more significant value in postoperative monitoring of LCA patients and tumor recurrence. After immunotherapy, a higher NLR was positively correlated with an increased risk of death in LCA patients, while a lower NLR was positively correlated with a longer overall survival period (60). Subsequently, based on the calculated cutoff value, the clinical correlation between LCA tumor features and postoperative immune therapy-related cytokines and immune subsets was assessed. Only IFN-γ showed statistical significance with distant tumor metastasis, while CD56^dim^ NK cells exhibited significant differences in lymph node infiltration. The other cytokines and cell subsets after post-LCA surgery immunotherapy do not show any clinical relevance. There are reports that IFN-γ can promote the metastasis of colorectal cancer through MACC1, so IFN-γ may promote the metastasis of LCA through some yet undefined mechanism (61). The results of a clinical correlation study on NK cells indicate that the distribution of CD56^dim^ NK cells is correlated with tumor metastasis, staging, and distribution (60). In addition, this study did not find any other cytokines or cell subsets related to the clinical features of LCA. Of course, there is currently controversy in related research, with some studies showing correlations while others do not, which will require further clinical studies for validation.

However, our study also has some limitations. Firstly, it is a retrospective study, and due to the rarity of checking cytokines and cell subsets in healthy populations, there are age differences between the enrolled LCA patients and the healthy control group. Secondly, because there are few LCA patients who are simultaneously tested for cytokines and cell subsets, the number of specimens included in the analysis in this study is relatively small. Finally, due to patient loss to follow-up, the survival data for some patients were lost.

In conclusion, we have confirmed that there are changes in cytokines and cell subsets in LCA patients after postoperative immunotherapy. Continuous monitoring of cytokines and cell subsets is more valuable than a single-time assessment. Furthermore, IL-6 and NLR, as two indicators, exhibit the strongest discriminatory ability between postoperative immunotherapy-treated LCA patients and healthy controls and should receive sufficient attention. The peripheral blood cytokines and immune cell profile have the potential value to become biomarkers for monitoring the immune status after LCA immunotherapy. For future research, we advocate designing prospective randomized controlled clinical trial protocols, further enriching the research cohort, exploring the changes in cytokines and cell subsets in patients with LCA after receiving targeted therapy, and their correlation with clinical characteristics of LCA, revealing their potential application in immune therapy monitoring after LCA surgery, and improving the survival rate and quality of life of LCA patients.

## Data availability statement

The original contributions presented in the study are included in the article/**Supplementary Material**. Further inquiries can be directed to the corresponding authors.

## Ethics statement

The application for the use of clinical data in this study was approved by the Ethics Committee of Shenzhen Luohu District People's Hospital (2023-LHQRMYY-KYLL-047). This study was a retrospective study, and written informed consent was not required.

## Author contributions

CZ: Data curation, Formal analysis, Methodology, Validation, Visualization, Writing – original draft. HM: Investigation, Project administration, Supervision, Writing – review & editing. ML: Data curation, Formal analysis, Writing – review & editing. SW: Methodology, Writing – review & editing. XD: Conceptualization, Funding acquisition, Project administration, Resources, Writing – review & editing. XZ: Conceptualization, Funding acquisition, Project administration, Resources, Supervision, Writing – review & editing.
