# Supplementary material for: The effects of postoperative targeted immunotherapy on peripheral blood cytokines and immune cell profile in lung cancer patients
Source: Front Oncol. 2024 Jun 5;14:1342624. doi: 10.3389/fonc.2024.1342624 (PMC11188683; doi:10.3389/fonc.2024.1342624)
Supplement: Supplementary file 1 [file DataSheet_1.docx]

**The effects of postoperative targeted immunotherapy on peripheral blood cytokines and immune cell profile in patients with lung cancer**

**Supplementary TABLE 1 Flow cytometry panels used for the identification of cell subsets**

| Number of tubes | Marker | Fluorochrome | Number of tubes | Marker | Fluorochrome |
| --- | --- | --- | --- | --- | --- |
| 1 | CD4 | FITC | 2 | CD57 | FITC |
|  | CD25 | PE |  | CD56 | PE |
|  | CD3 | ECD |  | CD3 | PC5 |
|  | CD8 | PC5 |  | CD16 | ECD |
| 3 | TCRγδ | FITC | 4 | CD11B | FITC |
|  | CD25 | PE |  | CD14 | PE |
|  | CD3 | ECD |  | CD16 | ECD |
|  | CD19 | PC5 |  | CD15 | PC5 |
|  |  |  |  | CD45 | PC7 |

**Supplementary TABLE 2 Calibration solution concentration dilution list**

Calibration Standard Preparation:

1. Prepare 8 centrifuge tubes as calibration standard gradient tubes, label them with numbers C0 to C7. Leave tube C7 empty. Add 50 μL of buffer solution to tube C1, and add 150 μL of buffer solution to the other 6 centrifuge tubes.
2. Add 0.5 mL of buffer solution to the calibration standard bottle, dissolve it thoroughly, mix well, and transfer it to tube C7 to create the highest concentration calibration solution.
3. Take 50 μL of the calibration solution from tube C7 and transfer it to tube C6, mix well. This is the 1:4 diluted calibration solution.
4. Subsequently, Transfer 50 μL of the calibration solution from tubes C6 to C2 successively to the next calibration gradient tube, and dilute stepwise to obtain calibration solution in tubes C5 to C1. The buffer solution in tube C0 serves as the calibration solution with a concentration of 0.

Please refer to the table below:

| Calibration product | Dilution ratio | Buffer volume | Standard product volume | Final concentration (pg/mL) |
| --- | --- | --- | --- | --- |
| C7 | -- | -- | -- | 5000 |
| C6 | 1:4 | 150μL | 50μL C7 | 1250 |
| C5 | 1:16 | 150μL | 50μLC6 | 312.5 |
| C4 | 1:64 | 150μL | 50μL C5 | 78.1 |
| C3 | 1:256 | 150μL | 50μL C4 | 19.5 |
| C2 | 1:1024 | 150μL | 50μL C3 | 4.9 |
| C1 | 1:4096 | 50μL | 50μL C2 | 2.4 |
| C0 | -- | 150μL | 0 | 0 |

**Supplementary TABLE 3 Comparison between peripheral Cytokines in living lung cancer patients and dying lung cancer**

|  | Living LCA patients (n=27) | |  | Dying LCA patients (n=25) | | *P*-value* |
| --- | --- | --- | --- | --- | --- | --- |
|  | Mean | SD |  | Mean | SD |  |
| IL-4 | 0.83 | 1.44 |  | 1.08 | 1.28 | 0.430 |
| IL-6 | 10.03 | 8.76 |  | 52.05 | 50.10 | **< 0.001** |
| IL-10 | 2.66 | 2.43 |  | 4.13 | 3.31 | **0.045** |
| IL-17 | 1.27 | 0.97 |  | 2.36 | 2.11 | 0.081 |
| IFN-γ | 2.22 | 2.73 |  | 10.80 | 14.34 | **0.006** |
| TNF-α | 1.29 | 2.23 |  | 2.51 | 3.87 | 0.097 |

* Mann-Whitney U test


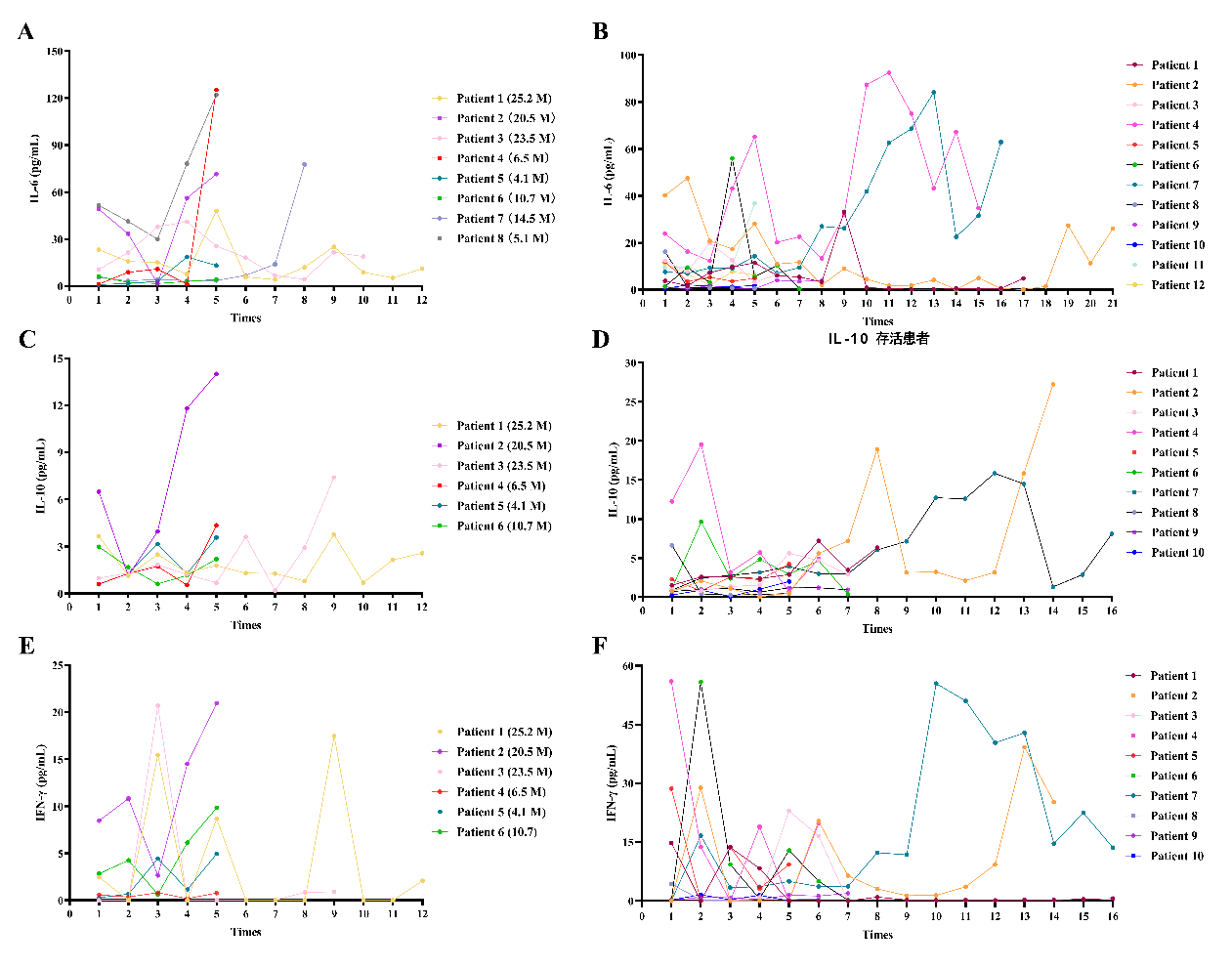


**Supplementary FIGURE 1 Continuous monitoring of cytokines in dying and living lung cancer patients**

**Supplementary TABLE 4 Comparison between peripheral blood immune cell profiles in living lung cancer patients and dying lung cancer**

|  | Living LCA patients (n=32) | |  | Dying LCA patients (n=25) | | *P*-value* |
| --- | --- | --- | --- | --- | --- | --- |
|  | Mean | SD |  | Mean | SD |  |
| CD3^+^CD4^+^CD8^+^ (%) | 0.42 | 0.57 |  | 0.30 | 0.24 | 0.784^U^ |
| CD3^+^CD25^+^ (%) | 6.86 | 7.45 |  | 7.13 | 12.64 | 0.125^U^ |
| CD3^+^CD4^+^CD25^+^ (%) | 10.18 | 5.46 |  | 9.98 | 6.58 | 0.717^U^ |
| CD3^+^CD56^+^ (%) | 3.93 | 3.58 |  | 4.21 | 2.10 | 0.169^U^ |
| CD3^-^CD56^+^CD16^+^ (%) | 96.02 | 3.25 |  | 89.90 | 10.16 | **0.005^U^** |
| CD3^+^TCRγδ^+^ (%) | 3.89 | 3.53 |  | 2.60 | 2.41 | 0.068^U^ |
| CD3^-^CD19^+^ (%) | 10.71 | 6.85 |  | 7.83 | 7.25 | **0.044^U^** |
| CD14^-^CD11B^+^ (%) | 62.15 | 10.52 |  | 76.26 | 8.76 | **< 0.001^T^** |
| CD14^+^ (%) | 8.37 | 3.48 |  | 8.00 | 3.83 | 0.479^U^ |
| CD14^+^CD15^+^ (%) | 10.77 | 13.42 |  | 13.82 | 10.86 | **0.035^U^** |
| CD14^+^CD16^+^ (%) | 31.35 | 24.14 |  | 28.44 | 16.35 | 0.748^U^ |
| NLR | 2.89 | 1.58 |  | 8.15 | 6.22 | **< 0.001^U^** |

* ^T^ Independent samples T test；^U^ Mann-Whitney U test


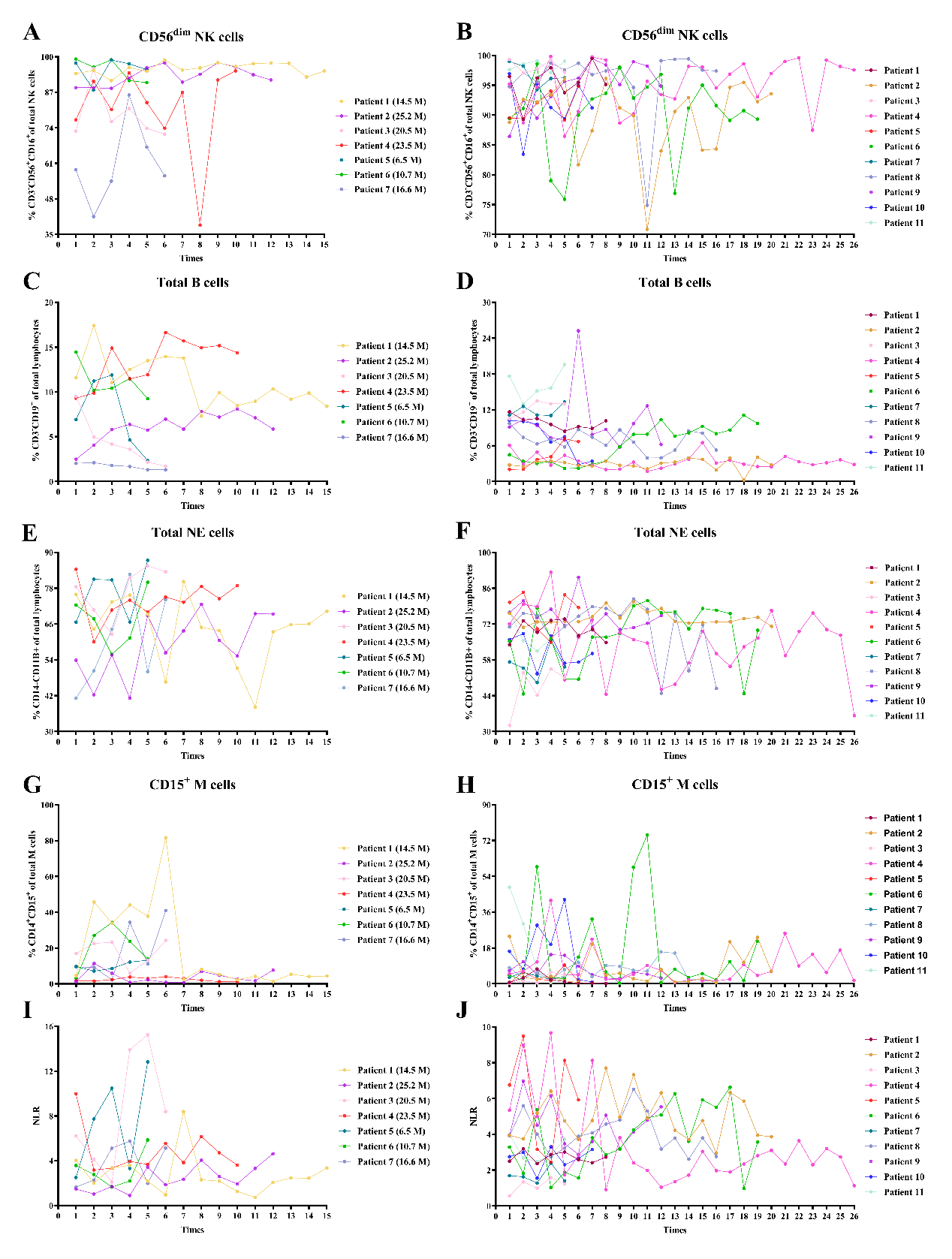


**Supplementary FIGURE 2 Continuous monitoring of peripheral blood immune cell profiles in dying and living lung cancer patients**

**Supplementary TABLE 5 Correlation analysis between cytokines and clinical characteristics of lung cancer patients**

|  | Factor | n | Positive diagnosis [n (%)] | Median | χ^2^ | *P-value* |
| --- | --- | --- | --- | --- | --- | --- |
| IL-6 | Tumor stage | | | | | |
|  | I-III | 12 | 9 (75.00) | 10.08 | 0.005 | 0.946 |
|  | IV | 54 | 41 (75.93) | 9.90 |  |  |
|  | Tumor differentiation grade | | | | | |
|  | Moderate | 22 | 18 (81.82) | 9.47 | 0.201 | 0.654 |
|  | Poor | 39 | 30 (76.92) | 10.08 |  |  |
|  | Lymph node invasion | | | | | |
|  | Yes | 57 | 44 (77.19) | 9.68 | 0.332 | 0.565 |
|  | No | 6 | 4 (66.67) | 9.47 |  |  |
|  | Distant metastasis | | | | | |
|  | Yes | 57 | 44 (77.19) | 9.90 | 0.242 | 0.623 |
|  | No | 10 | 7 (70.00) | 9.28 |  |  |
| IL-10 | Tumor stage | | | | | |
|  | I-III | 12 | 12 (100.00) | 2.05 | 0.946 | 0.331 |
|  | IV | 54 | 50 (92.95) | 1.71 |  |  |
|  | Tumor differentiation grade | | | | | |
|  | Moderate | 22 | 21 (95.45) | 1.71 | 0.010 | 0.919 |
|  | Poor | 39 | 37 (94.87) | 1.88 |  |  |
|  | Lymph node invasion | | | | | |
|  | Yes | 57 | 54 (94.74) | 1.88 | 0.332 | 0.565 |
|  | No | 6 | 6 (100.00) | 2.05 |  |  |
|  | Distant metastasis | | | | | |
|  | Yes | 57 | 53 (92.98) | 1.71 | 0.746 | 0.388 |
|  | No | 10 | 10 (100.00) | 2.05 |  |  |
| IFN-γ | Tumor stage | | | | | |
|  | I-III | 12 | 5 (41.67) | 2.09 | 0.570 | 0.450 |
|  | IV | 54 | 29 (53.70) | 2.39 |  |  |
|  | Tumor differentiation grade | | | | | |
|  | Moderate | 22 | 11 (50.00) | 2.39 | 0.233 | 0.629 |
|  | Poor | 39 | 22 (56.41) | 2.46 |  |  |
|  | Lymph node invasion | | | | | |
|  | Yes | 57 | 30 (52.63) | 2.46 | 0.015 | 0.902 |
|  | No | 6 | 3 (50.00) | 2.31 |  |  |
|  | Distant metastasis | | | | | |
|  | Yes | 57 | 31 (54.39) | 2.39 | **9.314** | **0.002** |
|  | NO | 10 | 3 (30.00) | 1.87 |  |  |

**Supplementary TABLE 6 Correlation analysis between immune cell and clinical characteristics of lung cancer patients**

|  | Factor | n | Positive diagnosis [n (%)] | Median | χ^2^ | *P-value* |
| --- | --- | --- | --- | --- | --- | --- |
| CD3^-^CD56^+^CD16^+^ (%) | Tumor stage | | | | | |
|  | I-III | 12 | 7 (58.33) | 95.47 | 0.031 | 0.861 |
|  | IV | 54 | 30 (55.56) | 95.28 |  |  |
|  | Tumor differentiation grade | | | | | |
|  | Moderate | 22 | 12 (60.00) | 95.28 | 0.284 | 0.594 |
|  | Poor | 39 | 24 (61.53) | 95.22 |  |  |
|  | Lymph node invasion | | | | | |
|  | Yes | 57 | 36 (63.16) | 95.22 | **4.841** | **0.028** |
|  | No | 6 | 1 (16.67) | 95.60 |  |  |
|  | Distant metastasis | | | | | |
|  | Yes | 57 | 30 (52.63) | 95.28 | 1.038 | 0.308 |
|  | No | 10 | 7 (70.00) | 95.70 |  |  |
| CD3^-^CD19^+^ (%) | Tumor stage | | | | | |
|  | I-III | 12 | 7 (58.33) | 7.20 | 0.166 | 0.684 |
|  | IV | 54 | 28 (51.85) | 7.39 |  |  |
|  | Tumor differentiation grade | | | | | |
|  | Moderate | 22 | 13 (59.09) | 7.75 | 0.041 | 0.839 |
|  | Poor | 39 | 22 (56.41) | 7.30 |  |  |
|  | Lymph node invasion | | | | | |
|  | Yes | 57 | 29 (50.88) | 7.49 | 2.547 | 0.110 |
|  | No | 6 | 1 (16.67) | 8.49 |  |  |
|  | Distant metastasis | | | | | |
|  | Yes | 57 | 30 (52.63) | 7.39 | 0.543 | 0.461 |
|  | No | 10 | 4 (40.00) | 8.49 |  |  |
| CD14^-^CD11B^+^ (%) | Tumor stage | | | | | |
|  | I-III | 12 | 10 (83.33) | 69.48 | 1.051 | 0.305 |
|  | IV | 54 | 37 (68.52) | 69.99 |  |  |
|  | Tumor differentiation grade | | | | | |
|  | Moderate | 22 | 17 (77.27) | 69.99 | 0.218 | 0.640 |
|  | Poor | 39 | 28 (71.79) | 70.41 |  |  |
|  | Lymph node invasion | | | | | |
|  | Yes | 57 | 43 (75.44) | 69.58 | 1.783 | 0.182 |
|  | No | 6 | 3 (50.00) | 71.19 |  |  |
|  | Distant metastasis | | | | | |
|  | Yes | 57 | 40 (70.18) | 69.99 | 0.404 | 0.525 |
|  | NO | 10 | 8 (80.00) | 66.82 |  |  |
| CD14+CD15+ (%) | Tumor stage | | | | | |
|  | I-III | 12 | 6 (50.00) | 6.16 | 0.345 | 0.557 |
|  | IV | 54 | 32 (59.26) | 6.16 |  |  |
|  | Tumor differentiation grade | | | | | |
|  | Moderate | 22 | 14 (63.64) | 5.78 | 1.026 | 0.311 |
|  | Poor | 39 | 22 (56.41) | 6.53 |  |  |
|  | Lymph node invasion | | | | | |
|  | Yes | 57 | 31 (54.39) | 5.78 | 1.857 | 0.173 |
|  | No | 6 | 5 (83.33) | 6.68 |  |  |
|  | Distant metastasis | | | | | |
|  | Yes | 57 | 36 (63.16) | 6.155 | 3.845 | 0.050 |
|  | NO | 10 | 3 (30.00) | 5.63 |  |  |
| NLR | Tumor stage | | | | | |
|  | I-III | 12 | 9 (75.00) | 3.58 | 0.627 | 0.429 |
|  | IV | 54 | 34 (62.96) | 3.62 |  |  |
|  | Tumor differentiation grade | | | | | |
|  | Moderate | 22 | 15 (68.18) | 5.78 | 1.026 | 0.311 |
|  | Poor | 39 | 26 (66.67) | 3.65 |  |  |
|  | Lymph node invasion | | | | | |
|  | Yes | 57 | 39 (68.42) | 3.58 | 0.829 | 0.363 |
|  | No | 6 | 3 (50.00) | 3.65 |  |  |
|  | Distant metastasis | | | | | |
|  | Yes | 57 | 37 (64.91) | 3.62 | 0.098 | 0.755 |
|  | NO | 10 | 7 (70.00) | 3.32 |  |  |
